# Cervical cancer screening varies by HPV vaccination status among a National Cohort of privately insured young women in the United States 2006–2016

**DOI:** 10.1097/MD.0000000000027457

**Published:** 2021-10-15

**Authors:** Djibril M. Ba, Jennifer S. McCall-Hosenfeld, Paddy Ssentongo, Vernon M. Chinchilli, Edeanya Agbese, Guodong Liu, Douglas L. Leslie, Ping Du

**Affiliations:** a Department of Public Health Sciences, Penn State College of Medicine, Hershey, PA; b Center for Applied Studies in Health Economics (CASHE), Penn State College of Medicine, Hershey, PA; c Department of Medicine, Penn State College of Medicine, Hershey, PA.

**Keywords:** cervical cancer screening, human Papillomavirus vaccine, private insurance, women

## Abstract

Human papillomavirus (HPV) vaccination in young women is low. Women aged 21 to 65 years in the United States (U.S.) have not reached the Healthy People 2020 objective of 93% for cervical cancer screening. The main aim of this study was to investigate the association between HPV vaccination status and cervical cancer screening among privately insured women aged 21 to 26 years in the U.S.

This was a retrospective cohort study using the IBM MarketScan database (2006–2016). The study population included 190,982 HPV-vaccinated women and 763,928 matched unvaccinated women. Adjusted incidence rate ratio (IRR) and the 95% confidence intervals (CIs) were obtained using the generalized estimating equations models with a Poisson distribution.

Among a total of 954,910 women included in the analysis, age (mean [SD]) was 23.3 [1.6] years. During 967,317 person-years of follow-up, a total of 475,702 incidents of cervical cancer screening were identified. The incidence density rates of cervical cancer screening were 461 per 1000 person-years (PY) for unvaccinated women and 787 per 1000 PY for those who received 3 doses of the HPV vaccine. After adjusting for other covariates, the IRR of cervical cancer screening was 34% higher among HPV-vaccinated women with at least one vaccine dose than unvaccinated women (adjusted IRR = 1.34, 95% CI: 1.33–1.35; *P* < .0001). The IRR of cervical cancer screening varied by the dose of HPV vaccination. There was evidence of a linear dose–response relationship between the number of HPV vaccine doses and cervical cancer screening (*P*-trend < .0001). Compared with unvaccinated women, the IRR of cervical cancer screening were 14%, 39%, and 60% higher among those who received 1, 2, and 3 doses of the HPV vaccine, respectively.

In this large retrospective cohort study of privately insured women, HPV-vaccinated women were more likely to be screened for cervical cancer compared with unvaccinated women.

## 1 Introduction

Human papillomavirus (HPV) is a known causal agent of cervical cancer and is also the most common sexually transmitted infection (STI) in the United States (U.S.).^[1]^ Sexually active women are at high risk of getting infected with HPV during their lifetime, with an estimated lifetime highest prevalence of 49.3% among those aged 20 to 24 years.^[2]^ In the U.S., it is estimated that 14,480 women will be newly diagnosed with cervical cancer, and 4290 will die from the disease in 2021.^[3]^

Globally, the introduction of the Papanicolaou (Pap) test for cervical cancer screening in the 1950s has contributed to a decrease in cervical cancer rates by >80%.^[4,5]^

Additionally, the HPV vaccine is a form of primary prevention of cervical cancer. It helps protect against high-risk HPV strains, responsible for 70% of cervical cancer cases and 90% genital warts cases.^[6,7]^ Currently, there are 3 Food and Drug Administration (FDA) approved HPV vaccines on the market—Gardasil (HPV genotype 6, 11, 16, and 18), Cervarix (HPV genotype 16, 18), and Gardasil 9 (HPV genotype 16, 18, 31, 33, 45, 52, and 58).^[8,9]^ Because of the importance of HPV vaccination to protect against infection with HPV and prevent HPV-associated cancers, in 2018, the President's Cancer Panel called for immediate action to increase HPV vaccine uptake as a national and international public health priority in the U.S. around the world.^[10]^ According to the International Papillomavirus Society, HPV vaccination, when used in combination with cervical cancer screening, would significantly decrease the burden of cervical cancer.^[11–14]^

In 2012, there was a consensus among professional organizations that issue cervical cancer guidelines recommending the adoption of cytology screening every 3 years for women aged 21 to 65 years and no screening for women younger than 21 years.^[15]^ However, a recent study by Watson et al^[16]^ showed women aged 21 to 65 years in the U.S. had not reached the Healthy People 2020 objective of 93% for cervical cancer screening. HPV vaccination remains below 50% among adolescents and young adults in the U.S.^[10,17–19]^

Previous studies that have examined the association between HPV vaccination uptake and cervical cancer screening have provided inconsistent findings. Studies conducted in Australia and Germany found no significant association between the uptake of the HPV vaccine and cervical cancer screening.^[20,21]^ However, a study conducted in Scotland, and 2 previous cross-sectional studies conducted in the U.S. using National Health Interview Survey data reported higher intention and uptake of cervical cancer screening among HPV vaccinated women.^[19,22,23]^ To the best of our knowledge, only a few studies have been published in the U.S. to examine how HPV vaccination uptake may affect cervical cancer screening behaviors among privately insured young adult women.^[22,23]^ Efforts are needed to better understand cervical cancer screening differences between women who received the HPV vaccine compared with those who did not receive the vaccine in the U.S.^[4]^ Therefore, the main aim of this study was to investigate the association between HPV vaccination status and cervical cancer screening. The second aim was to assess other predictors associated with cervical cancer screening among young women aged 21 to 26 years in the United States using the IBM MarketScan Research Database.

## 2 Materials and methods

### 2.1 Design and data source

This retrospective study analysis utilized data from the IBM MarketScan Commercial Database for the period January 1, 2006, through December 31, 2016, to assess factors associated with cervical cancer screening among women aged 21 to 26 years old.

The IBM MarketScan Commercial database include health insurance claims across the continuum of care (e.g., inpatient, outpatient pharmacy, etc) as well as enrollment data from large employers and health plans across the U.S. who provide private coverage for over million employees, their spouses, and dependents. This administrative claims database includes a variety of fee-for-service, preferred provider organizations, and capitated health plans.^[24]^ The IBM MarketScan databases are fully compliant with the U.S. privacy laws and regulations such as the Health Insurance Portability and Accountability Act (HIPAA). Patients’ demographic characteristics include information such as age, sex, and U.S. census regions. The protocol of this study was determined as a non-human subject research project by the Penn State College of Medicine Institutional Review Board. This study followed the Strengthening the Reporting of Observational Studies in Epidemiology (STROBE) reporting guideline for cohort studies.

### 2.2 Study cohort

A total of 954,910 young women aged 21 to 26 years were included in this analysis, of which 190,982 were vaccinated against HPV, and 763,928 were unvaccinated. Vaccinated women were identified using Current Procedural Terminology (CPT) codes from 2006 to 2016. Each vaccinated woman was randomly matched to 4 unvaccinated women (1:4) based on age, calendar year, and U.S. state of residency.

### 2.3 Assessment of outcome

The study outcome of interest was cervical cancer screening assessed during the follow-up period. The follow-up period was defined as at least 30 days after the index dates and ended at cervical cancer screening date, dis-enrollment date, death, or the end of the study period (December 31, 2016), whichever came first. Cervical cancer screening was identified using the 9th and 10th revision of the International Statistical Classification of Diseases (ICD-9 and ICD-10) and Current Procedural Terminology (CPT) **(**Table S1, Supplemental Digital Content**)**.^[25]^

### 2.4 Assessment of predictors of cervical cancer screening

We examined the following predictors to assess whether they were associated with cervical cancer screening: HPV vaccination status, age, place of residence (urban/rural), U.S. census regions, type of health plan, flu vaccine, previous Pap test, alcohol drinking, smoking, gonorrhea, chlamydia, syphilis, trichomoniasis, HIV/AIDS, Hepatitis B virus (HBV), Hepatitis C virus (HCV), depression, anxiety, and drug abuse. Previous studies reported that these variables above affect cervical screening.^[21,26]^ The following CPT codes were used to identify claim of HPV vaccine such as CPT codes 90649 (Gardasil), 90651 (Gardasil-9), and 90650 (Cervarix) among women aged 21 through 26 years from 2006 to 2016. Except for demographic variables, all the remaining predictors variables were assessed using ICD-9-CM, ICD-10-CM, and CPT codes **(**Table S2, Supplemental Digital Content**)**.

### 2.5 Statistical analysis

The statistical analyses were conducted using SAS version 9.4 software (SAS Institute, Cary, NC) and R software (R Foundation for Statistical Computing, Vienna, Austria) to generate figures. The index date for vaccinated women was defined as the earliest date of HPV vaccination. We assigned unvaccinated women the same index date as their corresponding matched vaccinated women. For each participant, person-years were calculated from the index dates to the first date of cervical cancer screening, end of enrollment, or end of the study period (December 31, 2016), whichever date came first. Descriptive analysis of cervical cancer screening status and predictor variables was conducted. The incidence density rate was calculated for each set of the predictors. The multivariable analysis was performed using generalized estimating equations (GEE; SAS Institute) with unstructured correlation structure, log link function, offset of log transformed follow-up, and Poisson distribution to explore the association between predictors and cervical cancer screening. To specify the use of the robust variance estimator for Poisson regression, the REPEATED statement was used (SAS GENMOD procedure).^[27]^ The results of the multivariable regression models are presented as the incidence rate ratios (IRR) and the 95% confidence intervals (CIs). The statistical tests were reported as significant if the significance level (*P*-value) was <.05 (2-sided).

## 3 Results

**Figure 1:**
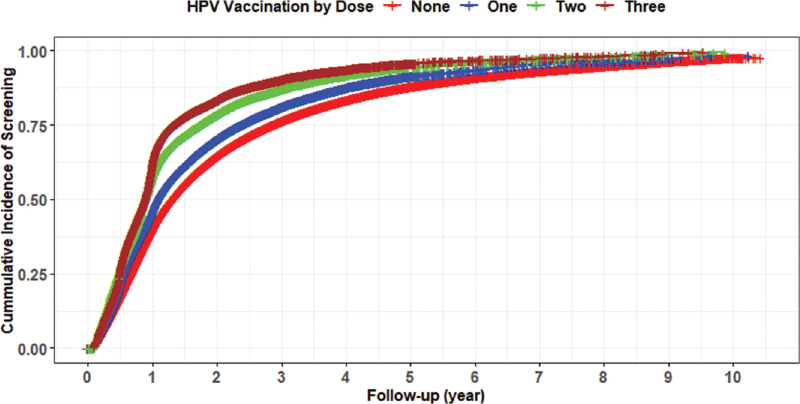
Graph illustrating the cumulative incidence of cervical cancer screening and HPV vaccination by dose. HPV = human Papillomavirus.

**Figure 2:**
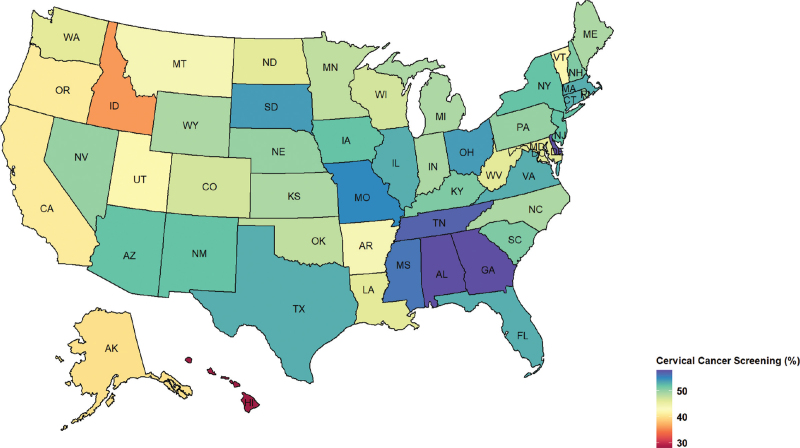
Cervical cancer screening rates (%) among privately insured women aged 21 to 26 years in the United States by the state during 2006 to 2016.

A total of 190,982 women HPV-vaccinated with at least 1 dose were identified with 763,928 matched unvaccinated women during 2006 to 2016. The mean age of the study sample was 23.3 (SD: 1.6) years. The cohort represented all 4 U.S. census regions with 35.7% from the South, 22.7% from Midwest, 22.5% from the West, and 19.2% from the Northeast. More than half one of the women had a preferred provider organization (PPO) health plan (58.6%). Most HPV-vaccinated women received 1 dose of HPV vaccine (44.9%), followed by 2 doses (28.3%) and 3 doses (26.8%), respectively. Gardasil quadrivalent was the most prevalent vaccine (95.5%), followed by Gardasil 9 (3.8%) and Cervarix (0.7%), respectively. During 967,317 person-years of follow-up, a total of 475,702 incidents of cervical cancer screening were identified. The overall cumulative incidence of cervical cancer screening during the study period was 49.8%. Figure 1 shows that women who received 3 doses had the highest cumulative incidence of cervical cancer screening followed by 2 doses, 1 dose, and 0 doses. Southern states of the U.S. had the highest cervical cancer screening rates, such as Georgia and Alabama, compared with other states (Fig. 2).

Table 1 shows cohort characteristics based on cervical cancer screening rates. The overall incidence density rate of cervical cancer screening among women who received at least 1 dose of HPV vaccine was 650 per 1000 PY during the study period. The incidence density rates of cervical cancer screening were 461 per 1000 person-years (PY) for unvaccinated women and 787 per 1000 PY for those who received 3 doses of the HPV vaccine. Furthermore, our results indicate that vaccinated women had higher levels of health care utilization prior to HPV vaccination initiation with the mean (SD) outpatient visit of 15.3 (25.4) compared with 4.0 (12.8) for unvaccinated women (data not shown). The incidence density rate of cervical cancer screening across ages range from 410 per 1000 PY for age 21 years to 591 per 1000 PY for 26 years. Similarly, the incidence rate of cervical cancer screening varied by type of health insurance plan, ranging from 453 per 1000 PY for CDHP/HDHP (consumer-driven health plan/high deductible health plan) to 510 per 1000 PY for those with a PPO health plan.

**Table 1:** Baseline characteristics of participants based on cervical cancer screening.

Characteristic	Total screened	Person-years (PY)	Screened IDR per 1000 PY
HPV vaccine status				
None	374,476	811,553	461
Received ≥1 vaccine	101,226	155,764	650
One dose	36,369	67,630	538
Two doses	28,397	41,814	679
Three doses	36,460	46,320	787
Age of the patients				
21	79,092	192,671	410
22	85,645	186,617	459
23	83,553	170,555	490
24	83,952	160,480	523
25	85,228	158,504	538
26	58,232	98,491	591
Place of residence				
Urban	428,098	869,406	492
Rural	47,604	97,911	486
US census region				
South	177,784	330,646	538
West	92,779	230,326	403
Midwest	110,519	215,502	513
Northeast	94,620	190,843	496
STDs				
Gonorrhea	231	374	618
Chlamydial	4976	7604	654
Syphilis	55	83	663
Trichomoniasis	392	658	596
HIV/AIDS	71	134	530
Hepatitis B virus	112	182	615
Hepatitis C virus	192	375	512
Non-STDs				
Flu vaccination	6497	11,446	568
Previous Pap test	91,752	136,383	673
Alcohol drinking	786	1506	522
Smoking	2404	4520	532
Drug abuse	496	1134	437
Depression	10,927	18,994	575
Anxiety	10,822	18,790	576
Type of health plan				
PPO	284,177	557,314	510
HMO	74,439	154,286	482
CDHP/HDHP	46,503	102,695	453
Others	70,583	153,023	461

Table 2 delineates the adjusted multivariable regression using GEE. Receipt of HPV vaccination, age, place of residence, U.S. regions, having chlamydia, receipt of flu vaccine, previous Pap test, smoking, drug abuse, anxiety, and type of health plans were predictors that were independently associated with cervical cancer screening. After adjusting for other covariates in the model, the IRR of cervical cancer screening was 6% higher among women with a history of chlamydial infection than those without chlamydial infection (adjusted IRR = 1.06, 95% CI: 1.03–1.10; *P* = .0001). Living in a rural area was associated with a 3% lower incidence rate of cervical cancer screening (adjusted IRR = 0.97, 95% CI: 0.96–0.98; *P* < .0001).

**Table 2:** Multivariable regression results using generalized estimating equations (GEE) with Poisson distribution modeling the predictors of cervical cancer screening.

Characteristics	Incidence rate ratio (IRR)	95% CI	*P*-value
HPV vaccine status			
None	Reference		
Received of ≥1 HPV vaccine	1.34	(1.33–1.35)	<.0001
One dose	1.14	(1.13–1.16)	<.0001
Two doses	1.39	(1.37–1.41)	<.0001
Three doses	1.60	(1.58–1.63)	<.0001
*P* for linear trend			<.0001
Age of the patients			
21	Reference		
22	1.13	(1.11–1.14)	<.0001
23	1.20	(1.18–1.21)	<.0001
24	1.29	(1.27–1.30)	<.0001
25	1.32	(1.31–1.34)	<.0001
26	1.41	(1.39–1.43)	<.0001
*P* for linear trend			<.0001
Place of residence			
Rural	0.97	(0.96–0.98)	<.0001
Urban	reference		
US census region			
South	Reference		
West	0.79	(0.78–0.80)	<.0001
Midwest	0.96	(0.95–0.97)	<.0001
Northeast	1.05	(1.04–1.06)	<.0001
STDs			
Gonorrhea	0.98	(0.84–1.15)	.81
Chlamydia	1.06	(1.03–1.10)	.0001
Syphilis	1.00	(0.72–1.39)	.99
Trichomoniasis	0.97	(0.87–1.08)	.61
HIV/AIDS	0.88	(0.67–1.14)	.33
Hepatitis B virus	1.11	(0.89–1.39)	.37
Hepatitis C virus	0.90	(0.77–1.07)	.23
Non-STDs			
Flu vaccination	0.94	(0.92–0.97)	.0001
Previous Pap test	1.22	(1.21–1.24)	<.0001
Alcohol drinking	0.97	(0.89–1.06)	.51
Smoking	0.95	(0.90–0.99)	.03
Drug abuse	0.87	(0.78–0.97)	.01
Depression	1.02	(0.99–1.04)	.24
Anxiety	1.03	(1.00–1.05)	.03
None	Reference		
Type of health plan			
PPO	Reference		
HMO	0.94	(0.93–0.95)	<.0001
CDHP/HDHP	0.93	(0.92–0.94)	<.0001
Others	0.90	(0.89–0.91)	<.0001

The IRR of cervical cancer screening was 1.34 times higher among HPV-vaccinated women with at least 1 vaccine dose than unvaccinated women (adjusted IRR = 1.34, 95% CI: 1.33–1.35; *P* < .0001). Similarly, the IRR of cervical cancer screening varied by the dose of HPV vaccination. There was evidence of a linear dose–response relationship between the number of HPV vaccine doses and cervical cancer screening (*P*-trend < .0001). Women who completed 3 HPV vaccine doses had the highest IRR of cervical cancer screening than unvaccinated (adjusted IRR = 1.60, 95% CI: 1.58–1.63; *P* < .0001). Women in the age group of 26 years had a 41% higher IRR of cervical cancer screening than the 21-year age group (adjusted IRR = 1.41, 95% CI: 1.39–1.43; *P* < .0001).

## 4 Discussion

In this large-scale, U.S. nationwide study of women with private insurance, we found a higher IRR of cervical cancer screening among HPV-vaccinated women than their unvaccinated counterparts. The cervical cancer screening rate in women who received at least 1 dose of the HPV vaccine was 34% higher than those who did not. This association was not confounded by age, geographic regions, comorbidities, and insurance type. Additionally, the rate of cervical cancer screening increased as the dose of HPV vaccination increased.

Our findings are consistent with previous studies conducted in the U.S. or other developed countries with different populations.^[19,22,23,26,28–33]^ More importantly, we observed a positive linear dose–response relationship between the number of HPV vaccine doses and cervical cancer screening. The incidence rate of cervical cancer screening was higher among women who received 3 doses of the HPV vaccine, followed by 2 doses and 1 dose. Our findings align with previous studies that found that the more vaccine doses a woman received, the higher the screening rate.^[26,30,32]^

It is plausible that HPV-vaccinated women's adherence to the recommended vaccine completion schedule may have contributed to adherence to routine cervical cancer screening. In addition, HPV vaccination is the primary prevention of cervical cancer, and cervical cancer screening is considered secondary prevention. Therefore, the observed association may be due to women's knowledge about cervical cancer-preventive behaviors. Furthermore, the administration of the HPV vaccine may be an educational event for health care providers to emphasize the need for continued cervical cancer screening, which may prompt increased screening uptake.^[26]^ The low rate of cervical cancer screening among unvaccinated women is concerning because many women remained unvaccinated and unscreened, resulting in increased cervical cancer risk in the future despite having private health insurance.

Although HPV vaccination provides an opportunity for the primary prevention of HPV infection among young women, cervical cancer screening plays a crucial role in detecting and treating HPV-associated diseases. A better understanding of women's cervical cancer screening behavior after HPV vaccination will inform prevention strategies to control cervical cancer and its related disability-adjusted life-years.^[34]^ Interestingly, vaccinated women also had higher levels of health care utilization prior to cervical cancer screening, suggesting greater access to care, higher general knowledge or awareness about preventive services, or more frequent interactions between health care providers that facilitate the decision to get screened for cervical cancer.

The present findings from our study revealed geographic heterogeneity in cervical cancer screening across states in the U.S., with the highest per-state rate of 58.0% (Georgia). Low cervical cancer screening rates in some states such as Hawaii and Idaho may be due to low HPV vaccination uptake. Women living in rural areas with limited access to healthcare or a high deductible health plans appeared to have lower cervical cancer screening rates than women with other health plans. Women with high deductible plans may avoid necessary health care services even though most high-deductible plans cover preventive care services with no out-of-pocket costs.

Furthermore, cervical cancer screening rates were decreasing in our study population from 2006 to 2016. Previous studies also found a similar trend in declining cervical cancer screening among 21 to 26-year-old women.^[16,25,35–37]^ As expected, younger women were less likely to undergo cervical cancer screening than women aged 26 years.^[22]^ Unexpectedly, receiving the flu vaccine, representing the general healthcare utilization behavior, was associated with a lower cervical cancer screening rate.

Findings from the present study suggested that HPV vaccine uptake remained low in the insured population and significant geographic variations exist in cervical cancer screening.^[10,38]^ Cervical cancer screening varied significantly between and within states of the U.S.

### 4.1 Study strengths and limitations

To our knowledge, this is the first large nationwide retrospective cohort study in the U.S. to investigate whether uptake of cervical screening differed by HPV vaccination status among privately insured young women aged 21 to 26 years. This study also benefited from a large sample size in a national claims database with the opportunity to identify general healthcare-seeking behaviors and geographic variations associated with cervical cancer screening. However, there are several limitations to be considered when interpreting these results. First, the study population is limited to patients with commercial private health insurance. Therefore, the findings may not be generalizable to non-privately insured populations, especially uninsured or underserved populations with higher risks for HPV-related cancers. Second, we may not be able to capture HPV vaccination history if women received vaccines elsewhere. Third, we acknowledge that claims-based databases can misclassify patients based on misreporting or underreporting of diagnoses using ICD-9-CM, ICD-10-CM, and CPT codes. Similarly, some variables such as smoking and drug abuse are underreported in insurance claims databases; thus, the prevalence of smoking and drug abuse may have been underestimated in this study. However, these errors due to misclassification may have a less significant effect with larger sample sizes. Finally, some critical covariates, such as racial/ethnic disparities, could not be considered for this study because of the lack of information in the MarketScan database. Despite these limitations, we believe this study provides new evidence regarding differences in cervical cancer screening behaviors between vaccinated and unvaccinated young women in the United States.

### 4.2 Clinical and public health implication

The combination of declining cervical cancer screening rates and low HPV vaccine uptake represents a critical challenge in cervical cancer prevention. Clinical and public health interventions are needed to increase both cervical cancer screening and HPV vaccination. The association between HPV vaccination uptake and cervical cancer screening suggests that vaccinated women are more likely to engage in health preventive behaviors. Therefore, healthcare providers will play an essential role in reminding women to receive cervical cancer preventive services about their HPV vaccination and cervical cancer during any clinical encounters.

## 5 Conclusion

HPV vaccination and cervical cancer screening rates remain low among privately insured young women, but receipt of at least 1 dose of HPV vaccination was associated with a higher rate of cervical cancer screening among U.S. young women. These results suggest that more intensive efforts are needed from public health professionals and healthcare providers to promote HPV vaccination uptake and cervical cancer screening at the national and state level.

## Author contributions

Designed research (project conception, development of overall research plan, and study oversight): Ping Du and Djibril M. Ba. Analyzed data or performed statistical analysis: Djibril M. Ba. All authors have read and approved the final manuscript.

**Conceptualization:** Djibril M. Ba, Douglas L. Leslie, Ping Du.

**Data curation:** Djibril M. Ba.

**Formal analysis:** Djibril M. Ba.

**Investigation:** Djibril M. Ba.

**Methodology:** Djibril M. Ba, Vernon M. Chinchilli, Guodong Liu, Douglas L. Leslie, Ping Du.

**Resources:** Djibril M. Ba, Vernon M. Chinchilli, Ping Du.

**Supervision:** Vernon M. Chinchilli, Douglas L. Leslie, Ping Du.

**Visualization:** Djibril M. Ba.

**Writing – original draft:** Djibril M. Ba.

**Writing – review & editing:** Jennifer S. McCall-Hosenfeld, Paddy Ssentongo, Vernon M. Chinchilli, Edeanya Agbese, Guodong Liu, Douglas L. Leslie, Ping Du.

## Supplementary Material

Supplemental Digital Content
