# Supplementary material for: Cervical cancer screening varies by HPV vaccination status among a National Cohort of privately insured young women in the United States 2006–2016
Source: Medicine (Baltimore). 2021 Oct 15;100(41):e27457. doi: 10.1097/MD.0000000000027457 (PMC8519251; doi:10.1097/MD.0000000000027457)
Supplement: Supplemental Digital Content [file medi-100-e27457-s001.docx]

| **Supplemental Table 1.** Diagnosis and Procedure Codes Used to Identify women Who Received Cervical Cancer Screening using the IBM® Watson Health MarketScan® database (2006-2016). | | | |
| --- | --- | --- | --- |
|  | **Coding System** | **Code** | **Code Description** |
| **Routine Gynecological Examination, Pelvic Examination or Papanicolaou (Pap) Test Encounter Codes** | | | |
|  | ICD-9-CM | V72.32 | Encounter for Papanicolaou cervical smear to confirm findings of recent normal smear following initial abnormal smear |
|  | ICD-9-CM | V76.2 | Routine cervical Papanicolaou smear |
|  | ICD-9-CM | V76.47 | Vaginal pap smear status-post hysterectomy for non-malignant condition |
|  | ICD-10-CM | Z12.4 | Encounter for screening for malignant neoplasm of cervix |
|  | ICD-10-CM | Z01.42 | Encounter for cervical smear to confirm findings of recent normal smear following |
|  | ICD-10-CM | Z12.72 | Encounter for screening for malignant neoplasm of vagina |
| **Pap Test Procedure Codes** | | | |
|  | HCPCS | G0101 | Cervical or vaginal cancer screening; pelvic and clinical breast examination |
| **Cervical or Vaginal Cytopathology codes** | | | |
|  | CPT-4 | 87620 | Papillomavirus, human, direct probe technique |
|  | CPT-4 | 87621 | Papillomavirus, human, amplified probe technique |
|  | CPT-4 | 87622 | Papillomavirus, human, quantification |
|  | CPT-4 | 87623 | Human Papillomavirus (HPV), low-risk types (e.g., 6, 11, 42, 43, 44) |
|  | CPT-4 | 87624 | Human Papillomavirus (HPV), high-risk types (e.g., 16, 18, 31, 33, 35, 39, 45, 51, 52, 56, 58, 59, 68) |
|  | CPT-4 | 87625 | Human Papillomavirus (HPV), types 16 and 18 only, includes type 45, if performed |
|  | CPT-4 | 88141 | Cytopathology, cervical or vaginal (any reporting system), requiring interpretation by physician |
|  | CPT-4 | 88142 | Cytopathology, cervical or vaginal (any reporting system), collected in preservative fluid, automated thin layer preparation; manual screening under physician supervision |
|  | CPT-4 | 88143 | Cytopathology, cervical or vaginal (any reporting system), collected in preservative fluid, automated thin layer preparation; with manual screening and rescreening under physician supervision |
|  | CPT-4 | 88147 | Cytopathology smears, cervical or vaginal; screening by automated system under physician supervision |
|  | CPT-4 | 88148 | Cytopathology smears, cervical or vaginal; screening by automated system with manual rescreening under physician supervision |
|  | CPT-4 | 88150 | Cytopathology, slides, cervical or vaginal; manual screening under physician supervision |
|  | CPT-4 | 88152 | Cytopathology, slides, cervical or vaginal; with manual screening and computer-assisted rescreening under physician supervision |
|  | CPT-4 | 88153 | Cytopathology, slides, cervical or vaginal; with manual screening and rescreening under physician supervision |
|  | CPT-4 | 88154 | Cytopathology, slides, cervical or vaginal; with manual screening and computer-assisted rescreening using cell selection and review under physician supervision |
|  | CPT-4 | 88164 | Cytopathology, slides, cervical or vaginal (the Bethesda System); manual screening under physician supervision |
| **Supplemental Table 1** (continued). | | | |
|  | **Coding System** | **Code** | **Code Description** |
| **Cervical or Vaginal Cytopathology Codes** (continued) | | | |
|  | CPT-4 | 88165 | Cytopathology, slides, cervical or vaginal (the Bethesda System); with manual screening and rescreening under physician supervision |
|  | CPT-4 | 88166 | Cytopathology, slides, cervical or vaginal (the Bethesda System); with manual screening and computer-assisted rescreening under physician supervision |
|  | CPT-4 | 88167 | Cytopathology, slides, cervical or vaginal (the Bethesda System); with manual screening and computer-assisted rescreening using cell selection and review under physician supervision |
|  | CPT-4 | 88174 | Cytopathology, cervical or vaginal (any reporting system), collected in preservative fluid, automated thin layer preparation; screening by automated system, under physician supervision |
|  | CPT-4 | 88175 | Cytopathology, cervical or vaginal (any reporting system), collected in preservative fluid, automated thin layer preparation; with screening by automated system and manual rescreening or review, under physician supervision |
|  | HCPCS | G0123 | Screening cytopathology, cervical or vaginal (any reporting system, collected in preservative fluid, automated thin layer preparation, screening by cytotechnologist under physician supervision |
|  | HCPCS | G0124 | Screening cytopathology, cervical or vaginal (any reporting system), collected in preservative fluid, automated thin layer preparation, requiring interpretation by physician |
|  | HCPCS | G0141 | Screening cytopathology smears, cervical or vaginal, performed by automated system, with manual rescreening, requiring interpretation by physician |
|  | HCPCS | G0143 | Screening cytopathology, cervical or vaginal (any reporting system), collected in preservative fluid, automated thin layer preparation, with manual screening and rescreening by cytotechnologist under physician supervision |
|  | HCPCS | G0144 | Screening cytopathology, cervical or vaginal (any reporting system), collected in preservative fluid, automated thin layer preparation, with screening by automated system, under physician supervision |
|  | HCPCS | G0145 | Screening cytopathology, cervical or vaginal (any reporting system), collected in preservative fluid, automated thin layer preparation, with screening by automated system and manual rescreening under physician supervision |
|  | HCPCS | G0147 | Screening cytopathology smears, cervical or vaginal, performed by automated system under physician supervision |
|  | HCPCS | G0148 | Screening cytopathology smears, cervical or vaginal, performed by automated system with manual rescreening |
|  | HCPCS | G0148 | Screening cytopathology smears, cervical or vaginal, performed by automated system with manual screening |
|  | HCPCS | Q0091 | Screening Papanicolaou (Pap) smear, obtaining, preparing and conveyance of cervical or vaginal smear to laboratory |
|  | HCPCS | P3001 | Screening Papanicolaou smear, cervical or vaginal, up to three smears requiring interpretation by a physician |
|  | HCPCS | P3000 | Screening Papanicolaou smear, cervical or vaginal, up to three smears, by a technician under the physician supervision |

**Abbreviations:** ICD-9-CM: International Classification of Diseases, 9^th^ ; ICD-10-CM: International Classification of Diseases, 10^th^ ; CPT: Current Procedural Terminology; HCPCS: Healthcare common procedure coding system

| **Condition** | **ICD-9-CM-ICD, ICD-10-CM, and CPT codes** |
| --- | --- |
| Gonorrhea | V02.7, 098, A54 |
| Chlamydial | 099.8, 099.9, 099.41, 099.50-099.59, V73.98, V73.88, 079.98, 077.98, 078.88, A74 |
| Syphilis | 091, 092, 094, 094-097, A51, A53 |
| Flu Vaccine | 90630, 90685, 90686, 90687, 90688, 90654, 90655, 90656, 90657, 90658  90660, 90661, 90662, 90653, 90666, 90667, 90668, 90664, 90672, 90673, 90674, 90756, 90682 |
| Trichomoniasis | 131, A59 |
| HIV/AIDS | 042, B20 |
| Hepatitis B | 070.20-070.23, 070.30-070.33, 070.42, 070.52, B16, B180, B181 |
| Hepatitis C | V02.60, V02.62, V02.69, V12.09, 070.41, 070.44, 070.51, 070.54, 070.70, 070.71,  B171, B182, B19.20 |
| Alcohol use | 291, 303, 305.0, 357.5, F10 |
| Smoking | V15.82, 305.1X, 649.0X, 989.84, F17.2, Z71.6, Z72.0, Z72.0, Z87.891, O99.33, Z87, Z87.8 or Z87.891. CPT codes: 99406, 99407, G0375, G0376, G0436, G0437, G8402, G8403, G8453, G8454, S4990, S4991, S4995, S9075, S9453, 4000F or 4001F |
| Drug abuse | 304, F19.10 |
| Depression | 296.2, 296.3, 296.5, 296.6, 300.4, 309.0, 309.1, 296.89, 311, F32, F33 |
| Anxiety | 300, F40-F48 |

**Supplemental Table 2. Codes for predictors assessment**

**Abbreviations:** ICD-9-CM: International Classification of Diseases, 9^th^ ; ICD-10-CM: International Classification of Diseases, 10^th^ ; CPT: Current Procedural Terminology; HCPCS: Healthcare common procedure coding system
